# Histology and PSMA Expression on Immunohistochemistry in High-Risk Prostate Cancer Patients: Comparison with ^68^Ga-PSMA PET/CT Features in Primary Staging

**DOI:** 10.3390/cancers15061716

**Published:** 2023-03-10

**Authors:** Luigia Vetrone, Riccardo Mei, Lorenzo Bianchi, Francesca Giunchi, Andrea Farolfi, Paolo Castellucci, Matteo Droghetti, Massimiliano Presutti, Alessio Degiovanni, Riccardo Schiavina, Eugenio Brunocilla, Antonietta D’Errico, Stefano Fanti

**Affiliations:** 1 Nuclear Medicine, Alma Mater Studiorum-University of Bologna, 40138 Bologna, Italy; 2 Division of Urology, IRCCS Azienda Ospedaliero-Universitaria di Bologna-Policlinico di Sant’Orsola, 40138 Bologna, Italy; 3 Pathology, IRCCS Azienda Ospedaliero-Universitaria di Bologna-Policlinico di Sant’Orsola, 40138 Bologna, Italy; 4 Nuclear Medicine, IRCCS Azienda Ospedaliero-Universitaria di Bologna-Policlinico di Sant’Orsola, 40138 Bologna, Italy

**Keywords:** PET-parameters, PSMA-immunochemistry, prostate cancer, PSMA PET

## Abstract

**Simple Summary:**

The aim of this study was to correlate primary staging prostate-specific membrane antigen (PSMA) positron emission tomography/computed tomography (PET/CT) parameters with either prostate final histopathology (pT) or PSMA-immunochemistry (IHC) features in a cohort of high-risk prostate cancer (PCa) patients. Our study demonstrated a correlation between higher SUVmax and IHC-PSMA expression. Moreover, we found correlations between higher total lesion PSMA (PSMA-TL) values and the risk of lymphovascular invasion (LVI), and between PSMA tumor volume (PSMA-TV) and the presence of a cribriform pattern. Our results, if validated by further studies, may help to better identify unfavorable features showed by PSMA-PET/CT in primary staging, thus improving patients’ management.

**Abstract:**

PSMA-PET/CT is a suitable replacement for conventional imaging in the primary staging of PCa. The aim of this retrospective study was to assess the correlation between parameters discovered by PSMA PET/CT in primary staging and either prostate histopathology (pT) findings or PSMA-IHC expression in a cohort of biopsy-proven high-risk PCa candidates for surgery. Clinical information (age, iPSA-value, and grading group) and PSMA-PET/CT parameters (SUVmax, PSMA tumor volume [PSMA-TV], and total lesion [PSMA-TL]) were compared with pT (including histologic pattern, Gleason grade, and lymphovascular invasion [LVI]) and PSMA-IHC features, including visual quantification (VS) with a four-tiered score (0 = negative, 1+ = weak, 2+ = moderate, 3+ = strong), growth pattern (infiltrative vs expansive), and visual pattern (cytoplasmic vs membranous). In total, 44 patients were enrolled, with a median age of 67 (IQR 57-77); the median iPSA was 9.4 ng/dL (IQR 12.5-6.0). One patient (3%) was grading group (GG) 3, 27/44 (61%) were GG4, and 16/44 (36%) were GG5. PSMA-PET/CT detection rate for the presence of primary prostate cancer was 100%. Fused/poorly formed Gleason grade 4 features were predominant (22/44–50%); a cribriform pattern was present in 18/44 (41%) and acinar in 4/44 (9%). We found that lower PSMA-TVs were mostly related to acinar, while higher PSMA-TVs correlated with a higher probability to have a cribriform pattern (*p*-value 0.04). LVI was present in 21/44(48%) patients. We found that higher PSMA-TV and PSMA-TL are predictive of LVI *p*-value 0.002 and *p*-value 0.01, respectively. There was no correlation between PET-parameters and perineural invasion (PNI), probably because this was present in almost all the patients. Moreover, patients with high PSMA-TL values displayed the highest PSMA-IHC expression (VS3+) with a membranous pattern. In conclusion, PSMA-TV and PSMA-TL are predictors of a cribriform pattern and LVI. These conditions are mostly related to higher aggressiveness and worse outcomes.

## 1. Introduction

Prostate-specific membrane antigen (PSMA) is a 100-kDa type II integral membrane protein, usually expressed in the cytoplasm of normal prostate tissue [1,2]. It is also particularly overexpressed in prostate cancer (PCa) cell membranes. Positron emission tomography (PET) targeting PSMA linked to either ^68^Ga or ^18^F has changed imaging approaches, improving the detection rate and diagnostic accuracy both in biochemical recurrence (BCR) and in primary staging before surgery or radiotherapy, with a particular focus on nodal localization and distant metastasis [3]. As demonstrated by a ProPSMA study [3], PSMA-PET/CT is a suitable replacement for conventional imaging, providing superior accuracy to the combined findings of CT and bone scanning in staging. In combination with an MRI, it improves the detection of primary PCa, avoiding unnecessary biopsies; it also seems to be more accurate in identifying PCa in patients with an iPSA > 4 ng/dL than an mpMRI alone [4]. Furthermore, it is a promising tool in patients with suspected PCa and a negative biopsy [4]. Moreover, the use of PSMA-PET/CT leads to a change in clinical management in approximately 28% of cases in primary staging [3] and 50% of cases in restaging [5,6]. However, the survival benefit of treatments changed based on PSMA-PET/CT is not yet fully assessed. Despite this, positive PSMA-PET/CT may be a prognostic tool for adverse outcomes and may be used for personalized PSMA-directed treatments [7,8], especially in BCR patients. Moreover, as Vision and TheraP suggested, PSMA-PET/CT is also necessary for the selection of patients with mCRPC that can benefit from ^177^Lu-PSMA therapy [9,10]. The likelihood of positivity for PSMA-PET/CT is influenced by several parameters, and different prediction models have been proposed to select patients who may benefit the most from PSMA-PET/CT imaging [11,12,13]. Nevertheless, as highlighted by immunohistochemistry studies, it is now known that there is a heterogenous PSMA expression in both primary tumors and distant metastasis [14]. Furthermore, around 10% of cases occur with low or without PSMA expression [15,16,17]. Interestingly, in these studies, most of the patients with low/absent PSMA expression showed infiltrative growth patterns. 

PSMA expression correlates with a higher tumor Gleason Score (GS) and with PCa progression; this is associated with increased DNA repair mutations and the development of hormone-resistant PCa [18,19]. We hypothesize that PSMA-PET/CT can provide semi-quantitative parameters that could be associated with peculiar histopathological features. The aim of this study was to assess the correlation between histology and immunohistochemistry (IHC) features at pathology and PSMA-PET/CT parameters in a cohort of high-risk PCa patients before radical prostatectomy (RP).

## 2. Background

### 2.1. Histopathological Parameters and Immunohistochemistry

#### 2.1.1. Histopathology

The majority of PCa (95%) cases are acinar adenocarcinoma, which is the fourth most common cancer and the eighth most common cause of cancer-related death in men. 

PCa Grade, according to the WHO Classification 2022 [20], is based on a grading group system (GGS) ranging from 1–5 and corresponds to the previous Gleason score. The new grading system recommends estimating the percentage and the type of pattern 4 in GG2 or GG3 in both needle biopsy and RP, as well as the presence of a cribriform pattern 4 in GG2 to GG4 due to the higher correlation with adverse clinical outcomes, including worse rates of biochemical recurrence-free outcomes, metastasis-free outcomes, and cancer-specific survival rates [21,22]. 

A feature that should be reported by pathologists is the presence of intraductal adenocarcinoma (IDC-P), particularly in the needle biopsy, because it is associated with a more aggressive PCa and with adverse pathological findings and clinical outcomes. In our analysis, we also focused our attention on lymphovascular (LVI) and perineural invasion (PNI). It is well known that tumor cells use lymphatic structures and vascular vessels to spread. That happens also for PCa. This is an unfavorable prognostic parameter and is correlated to a higher BCR risk. PNI is the presence of tumor cells around the nerve fibers. It represents a pathologic parameter suggestive of metastasis that can be detected in all tumors, including PCa. In these cases, tumor cells interact with the nerve components, creating a specific microenvironment that increases the cancer’s aggressiveness. In PCa cases, it is considered a negative prognostic value because it is associated with a high risk of BCR [23]. 

#### 2.1.2. Immunohistochemistry

In addition to histological evaluation, immunohistochemical staining is also widely used to corroborate the diagnosis of PCa. There are many different immunohistochemical panels used in order to differentiate the tumor versus normal glands: multiplex basal cell cocktails containing CKHMW [34βE12], p63, and p504s [AMACR]). These panels are also used to identify the origin of the cancer (NKX3.1, PSA, PSMA, and ERG) [24]. PSMA has a high sensitivity (88–100%) in distinguishing PCa from other urogenital tumors and in detecting metastasis of prostatic origin. 

## 3. Materials and Methods

### 3.1. Study Population

This is a single tertiary center retrospective study. We included all the 138 patients who underwent staging with ^68^Ga PSMA-PET/CT for high-risk PCa referred to our center from 1 April 2020 to 4 November 2021. We included only patients who underwent RP in our center; 86 patients were excluded for lacking data regarding radical prostatectomy. Eight patients were excluded because they were metastatic at presentation and surgery was not performed, leading to a final population of 44 patients. This study was approved by the local ethics committee (244/2016/O/Oss) and all patients gave written consent for the use of their data. All the information about clinical data (age of surgery, iPSA, tumor stage, WHO/ISUP prognostic grade group, and PSMA-PET/CT results) and pathological data (histology, ISUP, stage, nodal involvement, LVI, PNI, and immunohistochemistry) was collected.

### 3.2. Surgery

All patients underwent extended lymph node dissection (ePLND). The ePLND template included the fibrofatty tissue along the external iliac vein, with the distal limits being the deep circumflex vein and the femoral canal. Proximally, ePLND was performed up to and including the bifurcation of the common iliac artery. All fibrofatty tissue within the obturator fossa was removed to completely skeletonize the obturator nerve. The lateral limit consisted of the pelvic sidewall, and the medial dissection limit was defined by perivesical fat [25]. The dissection of presacral lymph nodes was performed in selected cases, according to the surgeon’s judgment and experience.

### 3.3. Histopathological Parameters and Immunohistochemistry

Prostate biopsies and radical prostatectomy (RP) specimens were fixed in formalin and embedded in paraffin. From paraffin blocks, 3-μm-thick sections were cut. The slides were stained with Hematoxylin- and eosin (H&E) using standard methods. PSMA IHC was conducted with automatic immunohistochemistry staining instrument Benchmark Ultra (Ventana/Roche Group 1910 Innovation Park, Tucson, Arizona, AZ 85755 USA). The antigen retrieval was conducted using Cell Conditioning 1 for 16 min at 99 °C, and the primary antibody PSMA (clone EP192, prediluted, Roche, Basilea, Switzerland) was incubated for 16 min at 36 °C. The revelation system used was OptiView DAB (12 min linker and 12 min HRP multimer) (Ventana/Roche, Basilea, Switzerland).

Histologic evaluation and PCa grading were performed by a dedicated genitourinary pathologist in accordance with criteria established by the World Health Organization’s WHO Classification of Tumors of the Urinary System and Male Genital Organs 2022. PCa growth (infiltrative vs expansive) and Gleason Grade group 4 pattern and percentage (poorly formed/fused glands, cribriform, glomeruloid, and ductal) were evaluated.

**Figure 1:**
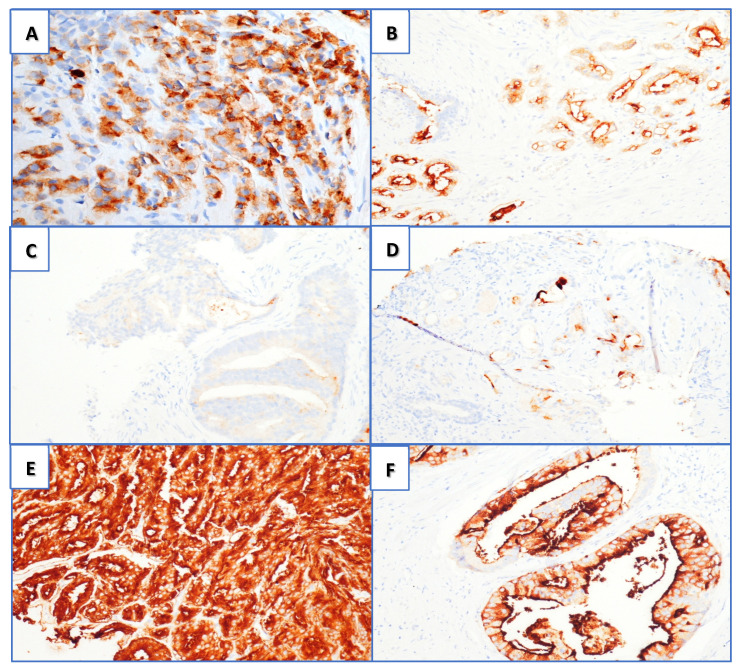
PSMA immunohistochemistry (magnification 20×): (**A**) cytoplasmatic immunoreaction; (**B**) membranous positivity. Visual score for PSMA positivity: (**C**) score 0, (**D**) score 1+, (**E**) score 2+, and (**F**) score 3+ (both cytoplasmic and membranous positivity).

PSMA IHC positivity as visual score (VS) was graded in a four-tiered system according to the intensity of the stain, as follows: 0 = negative, 1+ weak, 2+ moderate, and 3+ strong (Figure 1C–F). These were also divided according to membrane and/or cytoplasmatic expression (Figure 1A,B). We also evaluated the percentage of tumor-negative staining in the area of interest.

The PCa stage was assessed according to the American Joint Committee on Cancer (AJCC) cancer staging manual [26].

### 3.4. PET/CT Imaging and Analysis

**Figure 2:**
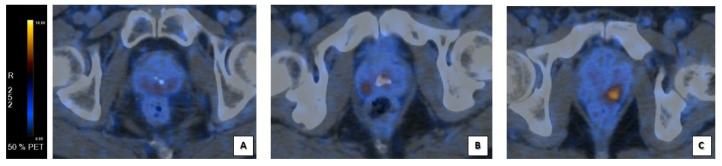
Corresponding visual score at PSMA-PET/CT scans’ analysis. Fusion images of the prostate gland: (**A**) faint diffuse PSMA uptake at prostate gland in VS1+ corresponding to image D in Figure 1 (**B**) focal PSMA uptake at the right lobe of prostate’s base in a VS2+ corresponding to image E in Figure 1; (**C**) focal, intense PSMA uptake at the left lobe of prostate’s base in a VS3+ corresponding to image F in Figure 1.

Patients underwent clinical routine ^68^Ga-PSMA-PET/CT on GE Discovery MI, GE Discovery STE, GE Discovery 710 (GE HealthCare Worldwide Milwaukee, WI, USA) after a single injection of ^68^Ga-PSMA-11 (mean dose 2 MBq/kg); low dose CT was acquired (with parameters of 120 KeV and 80 mA) according to the EANM procedure guidelines [27,28,29]. Dedicated workstations (AW Server 3.2 Ext. 2.0 GE Healthcare Worldwide Milwakee) were used to examine PSMA-PET/CT images, allowing simultaneous and fused review of PET and CT data. Each scan was blindly reviewed for any clinical information. The image analysis was based on the visual identification of areas with significant PSMA uptake, defined by comparison to background uptake (Figure 2). After finding the lesion’s area, a spherical volume of interest (VOI) was drawn in order to include the entire lesion, and SUVmax was automatically measured in the VOI. Total Volume (PSMA-TV) and PSMA total lesion (PSMA-TL) of the localization of PCa in prostate gland were collected as semi-quantitative parameters, used to provide quantitative imaging biomarkers to assess the tumor burden [30,31]. PSMA-TV was semi-automatically calculated in cubic centimeters. PSMA-TL was semi-automatically calculated as the product of PSMA-TV and SUVmean. PSMA SUVmax, Total Volume (PSMA-TV), and PSMA total lesion (PSMA-TL) were collected with a SUVmax threshold of 40% within the lesion.

### 3.5. Statistical Analysis 

Data were analyzed using SPSS Statistics (IBM, v.26). Mann–Whitney’s U Test was used to assess the correlation between LVI at pathologic specimen and PSMA-PET-parameters, iPSA, and % T neg. The same analysis was performed for the correlation of PET-parameters with perineural invasion, visual pattern, and visual growth at pathologic specimen. Kruskall–Wallis test was used to correlate PET-parameters with different histologic patterns and visual score at pathologic specimen. Contingency table was performed to assess the correlation between perineural invasion and visual score at pathologic specimen. Statistical significance was reached with *p*-values < 0.05.

## 4. Results

### 4.1. Study Population

In total, 44 patients were analyzed with a median age of 67 (IQR 57-77); median iPSA was 9.4 ng/dL (IQR 12.5-6.0). At the final pathology, one (3%) patient was GG3, 27/44 (61%) GG4, and 16/44 (36%) GG5 (Table 1).

### 4.2. Surgery

Positive surgical margins (R1) were found in 20/44 (45%) patients and negative surgical margins were found in 24/44 (55%) patients. Positive lymph nodes at histopathological analysis (pN1) were found in 13/44 (30%); the others, 31/44, (70%) were negative. 

### 4.3. Histopathological Parameters and Immunohistochemistry

At RP evaluation, 1/44 (2.3%) patients were 100% acinar carcinoma, 4/44 (9.0%) were 100% cribriform, 9/44 (20.5%) were 100% fused, and 30/44(68.2%) were mixed-pattern histotypes (acinar/fused/cribriform). No intraductal PCa was found, neither glomeruloid nor ductal Gleason grade 4 pattern. The mixed histologic types were stratified according to the prevalent histotype as follows: 4/44 (9%) acinar carcinomas, 18/44 (41%) cribriform, and 22/44 (50%) fused (Table 1; Table 2). At the final pathologic specimen of RP, as showed in Table 2, the IHC analysis concerning PSMA expression was recorded as follows: VS 3+ was present in 35/44 patients (79.5%), VS 2+ in 6/44 (13.7%), and VS 1+ in 3/44 (6.8%). All the 4/4 (100%) acinar PCa patients were VS3+, with 2/4 (50%) having an expansive growth pattern and 2/4 (50%) having an infiltrative growth pattern; 2/4 (50%) had a cytoplasmatic expression, 1/4 (25%) had a membranous expression, and 1/4 (25%) had a mixed expression (membranous + cytoplasmatic). Overall, 15/18 (83.3%) cribriform histotypes were VS3+ and 3/18 (16.7%) were VS2+; 15/22 (68.2%) fused-form histotypes were VS3+, 3/22 (13.6%) were VS2+, and 4/22 (18.2%) were VS1+ (Table 3). An infiltrative growth pattern was present in 27/44 (61.4%) and expansive in 17/44 (38.6%). A membranous PSMA expression was present in 10/44 (22.7%) while cytoplasmatic expression was present in 12/44 (27.3%) and a mixed pattern was present in 22/44 (50.0%) (Table 2). Finally, a correlation between visual score and visual pattern is reported in Table 4. 

### 4.4. Imaging

All the scans were positive for uptake in the prostate gland. The median SUVmax value for pT was 19.5 (IQR 21.6–8.0). 

Overall, 29/44 (65.9%) patients had a multifocal localization on the prostate gland; 15/44 (34.1%) had a unique focus. The median PSMA-TV was 4.5 (IQR 10.7–2.2). The median PSMA-TL was 35.9 (IQR 49.7–14.3). Both the PSMA-TV and the PSMA-TL were measured only on the tumor localization on the prostate gland. The intrapelvic lymph nodes’ involvement in PSMA-PET/CT images was present in 8/44 (18%). The extra-pelvic lymph nodes’ involvement was present in one patient. 

Distant bone metastasis (M1) was detected by PSMA-PET7CT in 8/44 patients, who were excluded from surgery. There were no single bone localizations; 4/8 (50%) were oligometastatic and 4/8 (50%) were multi-metastatic. Additionally, 1/8 had a single lung lesion. 

### 4.5. Correlations

#### 4.5.1. Lymphovascular Invasion and PET-Parameters

**Figure 3:**
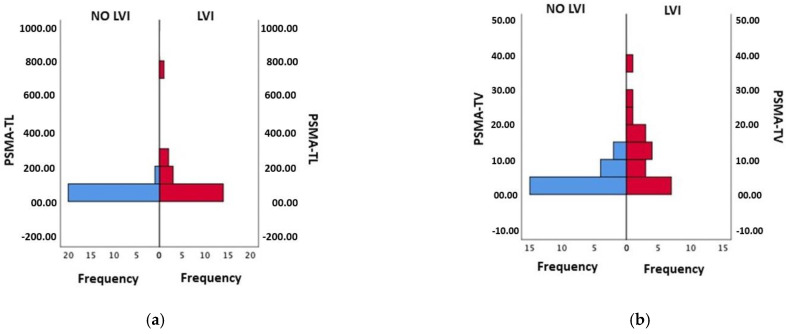
(**a**) Mann–Whitney Test for PSMA-TL and LVI (*p*-value 0.01); (**b**) Mann–Whitney Test for PSM-TV and LVI (*p*-value 0.002).

LVI was present in 20/44 (45%) patients. Using Mann–Whitney’s U Test, we found a correlation between the PSMA-TV and the PSMA-TL with LVI at the final pathology (all *p*-values ≤ 0.01). Moreover, patients with a higher iPSA had LVI (*p*-value = 0.025) (Figure 3). There was no significant correlation between LVI at the final pathology and SUVmax, or with T%neg.

#### 4.5.2. Perineural Invasion and PET-Parameters

Overall, 41/44 (93%) had PNI. No significant correlation was found between PNI and PET parameters (SUVmax, PSMA-TV, and PSMA-TL), iPSA, and T%neg. Nevertheless, at pT analysis, PNI correlated with the visual score: it was present in 35/35 (100%) VS 3+, 4/4 (100%) VS 2+, and 3/4(75%) VS 1+. 

#### 4.5.3. Histology and PET-Parameters

**Figure 4:**
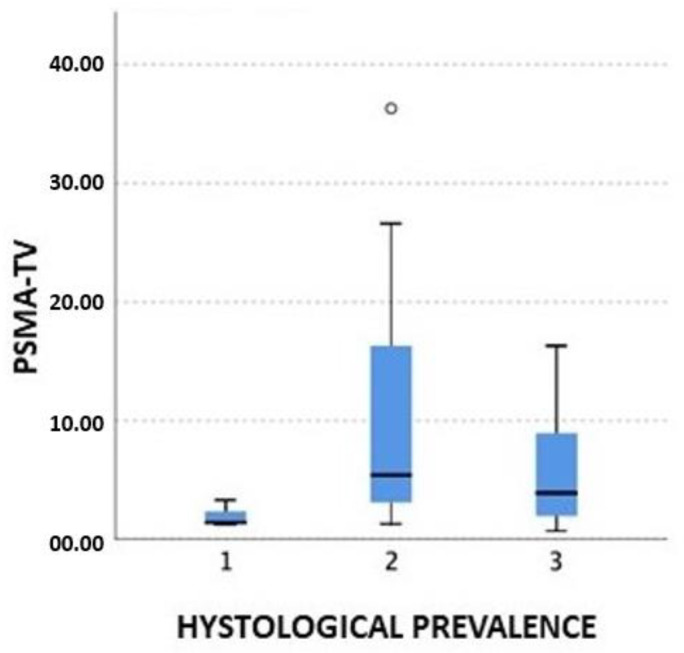
PSMA-TV and histological prevalence (1: acinar; 2: cribriform; 3: fused/poorly).

The Kruskall–Wallis test showed a significant correlation between the PSMA-TV and the histology: the higher the PSMA-TV, the higher presence of a cribriform pattern at pT, while the acinar showed to have a lower volume at presentation, even in high-risk PCa cases (*p*-value 0.04) (Figure 4). 

#### 4.5.4. Immunohistochemistry and PET-parameters

By comparing PSMA PET parameters, we found a correlation between high PSMA-TL values and PSMA expression on IHC (*p* = 0.025), especially when considering VS 1+ vs. 2+ and VS 1+ vs. VS 3+. Furthermore, we identified a strong correlation between increased SUVmax and VS 3+ (*p*. value 0.03). PSMA-TL was correlated with the visual pattern (membranous or cytoplasmic): the higher the PSMA-TL, the higher presence of the membranous pattern (*p*-value 0.009). Moreover, SUVmax increased in patients with membranous patterns (*p*. value 0.03) as compared with patients without membranous patterns. There was no significant correlation between any PET-parameters and visual growth pattern (infiltrative vs. expansive). 

Results are summarized in Table 5. 

## 5. Discussion

Not many studies have investigated the specific correlations between PSMA PET parameters and histopathology [15,17]. Rüschoff et al. [17] studied the specific characteristics that prevent a negative PSMA-PET/CT. They identified the percentage of negative tumor area, infiltrative growth pattern, smaller tumor size, and ISUP grade 2 as prognostic of lower 68Ga-PSMA-PET/CT uptake. We focused our attention, instead, on finding correlations among PSMA PET parameters, histopathology, and immunochemistry in a cohort of high-risk PCa patients in order to identify features that can correlate with histopathology. We found that the PSMA-TV and the PSMA-TL are predictive of LVI and that higher PSMA-TV values display a cribriform pattern. 

### 5.1. Lymphovascular and Perineural Invasion

Our data showed that LVI correlates with higher PSMA-TV, PSMA-TL, and iPSA values. LVI is a negative prognostic finding related to unfavorable outcomes, reduced BCR-free survival, and increased metastatic risk. In fact, as Shifaa et al. [32] demonstrated, 54% of LVI-positive cases showed seminal vesicle invasion (pT3b) and 28% had pN1 at RP. In addition, Gesztes et al. [33] showed that there is a significant association between LVI and p53 expression; they subdivided PCa patients in p53+ and p53- groups and found that 39.8% of group 1 was LVI positive. Multiple studies have reported the correlation between IHC p53 expression and PCa progression. In fact, it is well known that p53 gene mutations lead to unregulated cellular growth with many malignancies, including PCa [34]. It has been demonstrated since 1995 that p53 expression in PCa cells is an independent prognostic factor of disease progression after RP [35]. PSMA is well known to be expressed in the neoangiogenesis process, necessary to tumor growth. Maybe there is a correlation between p53 and PSMA expression. Due to the negative prognostic value of LVI, especially for BCR-free survival, PSMA PET parameters, particularly PSMA-TL, might be useful in the future as an additional tool for selecting patients for cancer treatment intensification. Further studies with larger populations and with oncologic follow-up data are needed in order to validate this hypothesis and maybe to find a specific cut-off value as a predictor of LVI. 

Perineural invasion is represented by the interaction between the cancer cells and the nerves’ fiber. It is a common process in PCa. In our cohort, 41/44 of the RP specimens showed PNI. This is the reason why our results did not show any correlation between PET parameters (PSMA-TV, PSMA-TL, and SUVmax) and PNI.

### 5.2. PSMA Immunochemistry on pT

PSMA expression was seen in all (100%) of the RP specimens. IHC VS is also correlated with SUVmax on 68Ga-PSMA PET/CT. We did not find a specific cut-off value predictive of higher PSMA expression on IHC analysis, probably because of the small study population. Further investigations are needed to assess whether higher PSMA expression (VS 3+) in IHC analysis might be related to an increased risk of early BCR and metastasis after RP. 

Moreover, the SUVmax and the PSMA-TL are also related to the membranous PSMA expression. As demonstrated by Paschalis et al. [36], membranous PSMA expression is connected to higher GG and a worse overall survival rate since it is also related to defective DNA damage repair. 

### 5.3. Histopathology

Our analysis showed that PSMA-TV is higher for the cribriform histotype and lower for the acinar histotype. The incidence of a cribriform pattern is about 25–34% of prostate biopsies [37]. It is important to report the cribriform morphology due to its impact on patient management. In fact, it is well known that the cribriform pattern is correlated with a higher BCR rate and associated with a higher metastatic risk. Hong Yuen Wong et al. [37] demonstrated that there is a specific interaction between the cancer cells in the cribriform pattern and the tumoral microenvironment; they showed the presence of MYC-induced genes’ upregulation and a strong enrichment of TNFa signaling. There is also a higher expression of SCHLAP1, a long non-coding RNA associated with the progression to metastasis. The PSMA-TV could be useful to predict the presence of a cribriform pattern at the final pathology and may be an important tool for the identification of patients at higher risk of recurrence after primary treatment, who may need additional treatments or follow-up intensification.

PCa growth pattern is classified as infiltrative (INF) or expansive (EXP) according to the presence of gross tumoral masses or infiltrative tumor cells among benign glands. Laudicella et al. [38] showed that the INF growth pattern has lower SUVmax at PSMA-PET/CT compared to the EXP growth pattern, and that the EXP pattern is associated with a higher ISUP. However, in our cohort, we had 17/44 patients exhibiting the EXP pattern and 27/44 patients exhibiting the INF pattern, and we did not find any significant correlation between the PET parameters and the growth pattern. Different results could be due to the inclusion in our cohort of only high-risk patients with a higher PSMA expression at PSMA-PET/CT; this may explain the lack of discrimination between EXP and INF patterns by PET parameters.

### 5.4. Limitation

Despite several strengths, our study is not devoid of limitations. First, the retrospective design of the study may have influenced the selection process of our cohort. Second, the limited number of patients included may have influenced the final results. Thus, the cohort should be enlarged for further analysis. Third, it was impossible to assess the impact of histology, IHC, and PSMA-PET parameters on the patient’s outcome. 

## 6. Conclusions

Our study showed that a higher IHC PSMA expression at the final pathology correlates with a higher SUVmax, and there is a significant correlation between PET/CT PSMA-TL, PET/CT PSMA-TV and LVI, and between PET/CT PSMA-TV and a cribriform pattern, which are known to be strong predictors of a higher risk of progression and metastases in PCa patients. Further studies with larger populations and a longer follow-up are needed to identify and better assess their prognostic value.

## Figures and Tables

**Table 1:** Population’s characteristics.

DATA	MEDIAN	IQR	%
Age iPSA GG3 GG4 GG5	67 years old	57–77	
	9.4 ng/dL		
			3% 61% 36%
pT acinar			9%
pT cribriform			41%
pT fused/poorly			50%

**Table 2:** Pathology features’ results.

PATHOLOGY FEATURES	PATTERN	PERCENTAGE
**HISTOTYPE**	pT Acinar	9%
	pT Cribriform	41%
	pT Fused/Poorly	50%
	pT Intraductal	0%
	pT Glomeruloid	0%
	pT Ductal	0%
**VISUAL SCORE**	VS 1+	6.8%
	VS 2+	13.7%
	VS 3+	79.5%
**VISUAL PATTERN**	Cytoplasmic	27.3%
	Membranous	22.7%
	Mixed	50%
**VISUAL GROWTH**	Infiltrative	61.4%
	Expansive	38.6%

**Table 3:** Correlation between visual score and histological results.

VISUAL SCORE	HYSTOLOGICAL PATTERN	N %
**VS3+**	fused/poorly formed cribriform	16/35 45.7% 15/35 42.8% 4/35 11.4%
	
**VS2+**	Cribriform Fused/poorly	3/6 50% 3/6 50%
**VS1+**	Fused	3/3 100%
	

**Table 4:** Correlation between visual score and visual pattern.

VISUAL SCORE	VISUAL PATTERN	N %
**VS3+**	Membranous Cytoplasmic Mixed	3/35 8.6% 11/35 31.4% 21/35 60%
**VS2+**	Membranous Cytoplasmic Mixed	4/6 66.6% 1/6 16.7% 1/6 16.7%
**VS1+**	Membranous	3/3 100%

**Table 5:** Correlations among PET-parameters and histopathology features.

	pT- SUVmax	PSMA-TV	PSMA-TL
**HYSTOTYPE**	/	Higher values are related to cribriform	/
		(*p*-value 0.04)	
**VISUAL SCORE**	Higher values displayed VS 3+	/	Higher values displayed VS 3+
	(*p*-value 0.03)		(*p*-value 0.025)
**VISUAL PATTERN**	/	/	Membranous
			(*p*-value 0.009)
**VISUAL GROWTH**	/	/	/
**LVI**	/	Predictive value (*p*-value 0.002)	Predictive value (*p*-value 0.01)
**PNI**	/	/	/

## Data Availability

The data supporting this study’s findings are available on request from the corresponding author.
